# Smells like home: Desert ants, *Cataglyphis fortis*, use olfactory landmarks to pinpoint the nest

**DOI:** 10.1186/1742-9994-6-5

**Published:** 2009-02-27

**Authors:** Kathrin Steck, Bill S Hansson, Markus Knaden

**Affiliations:** 1 Department of Evolutionary Neuroethology, Max Planck Institute for Chemical Ecology, Hans Knoell Strasse 8, 07745 Jena, Germany

## Abstract

**Background:**

*Cataglyphis fortis *ants forage individually for dead arthropods in the inhospitable salt-pans of Tunisia. Locating the inconspicuous nest after a foraging run of more than 100 meters demands a remarkable orientation capability. As a result of high temperatures and the unpredictable distribution of food, *Cataglyphis *ants do not lay pheromone trails. Instead, path integration is the fundamental system of long-distance navigation. This system constantly informs a foraging ant about its position relative to the nest. In addition, the ants rely on visual landmarks as geocentric navigational cues to finally pinpoint the nest entrance.

**Results:**

Apart from the visual cues within the ants' habitat, we found potential olfactory landmark information with different odour blends coupled to various ground structures. Here we show that *Cataglyphis *ants can use olfactory information in order to locate their nest entrance. Ants were trained to associate their nest entrance with a single odour. In a test situation, they focused their nest search on the position of the training odour but not on the positions of non-training odours. When trained to a single odour, the ants were able to recognise this odour within a mixture of four odours.

**Conclusion:**

The uniform salt-pans become less homogenous if one takes olfactory landmarks into account. As *Cataglyphis *ants associate environmental odours with the nest entrance they can be said to use olfactory landmarks in the vicinity of the nest for homing.

## Background

As a result of its amazing navigational capabilities, the desert ant *Cataglyphis fortis *has become a model organism for studying orientation [1-4]. In search of food, individual ants depart on tortuous routes often leading them more than 100 m from the nest. Once they find a food item, the ants return directly to the inconspicuous nest entrance. The ability to navigate so precisely has so far been thought to result from two synergistic visual systems. For long-distance navigation, the ants perform path-integration, getting the direction of movement by a skylight compass [1,2] and the distance by a step integrator [5]. Owing to the egocentric nature of this kind of orientation, errors may accumulate during the forage run. Therefore, as soon as the path integrator has guided the ants to the vicinity of the nest, they shift their attention to visual landmarks in the immediate surroundings of the nest [3,4]. Hence, the large-eyed *Cataglyphis *has been deemed a typical vision-guided insect. The role of olfaction has so far been considered to be restricted mainly to nest mate recognition [6,7] and to the localization of food [8]. Food is usually distributed randomly as the ants forage for dead arthropods [9]. Furthermore, route pheromones used by the ants would be rather short-lived given the hot ground – up to 60°C – of the salt-pan. Both the unpredictable food distribution and the high surface temperature might account for the fact that mass recruitment and orientation along odour trails have never been observed in members of the genus *Cataglyphis *[10]. Here we found location-specific blends in the salt-pans that differed in their composition of single-odour components. As the varying components showed electroantennogram (EAG) activity in *Cataglyphis*, they represent potential olfactory landmarks that the ants could use as cues for fine-scale navigation.

Furthermore, we show that *Cataglyphis *ants use environmental olfactory information for homing. We designed an experimental paradigm in which the ants associated a visually inconspicuous nest entrance with a given monomolecular odour. Not only did the ants learn to associate the nest with the training odour, but they were also able to distinguish this odour from a set of non-training odours. Hence, *Cataglyphis *ants are able to learn odours in the vicinity of the nest entrance and use these odours to pinpoint the nest during homing.

## Results

### Potential olfactory landmarks are present in the salt-pan habitat

**Figure 1:**
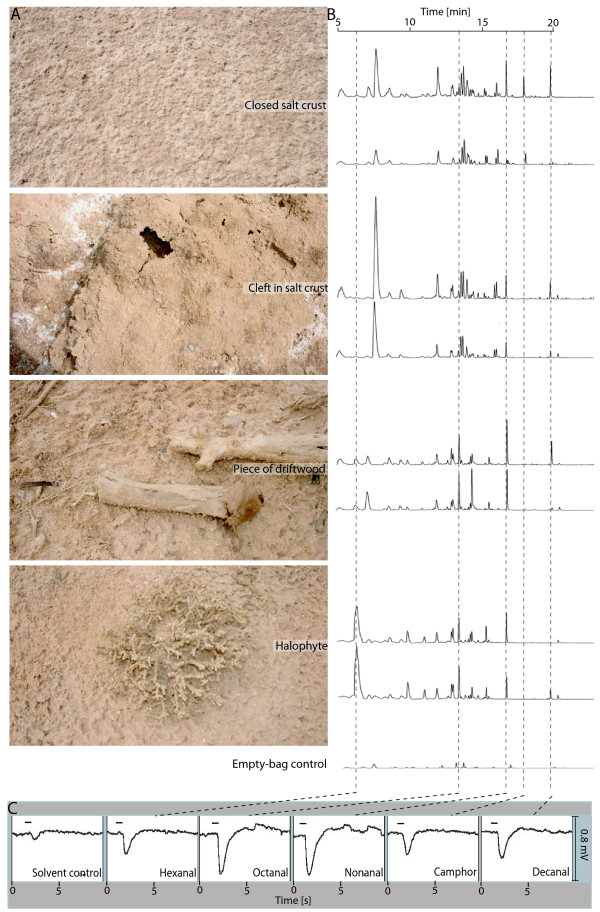
**Olfactory landmarks in the *Cataglyphis *habitat**. A. Sample locations. B. Location-specific gas chromatographic profiles collected on consecutive days are displayed next to the corresponding photo. Dashed lines depict identified components that were used for EAG recordings. C. EAG responses of *Cataglyphis *to the identified components. Horizontal bars indicate the stimulus duration.

Despite its homogenous appearance, the flat ground within the salt-pan habitat differs slightly in its soil structure. Covered by a continuous salt crust, the surface is occasionally interrupted by clefts or by pieces of wood and halophytic plants, signs of past periods of flooding (Figure 1A). In order to check whether these structures result in different habitat odours, we used gas chromatography to analyse headspace samples of continuous salt crust, cleft salt crust, wood and halophytic plants. The emitted volatiles for each sample were relatively constant over two consecutive days, whereas the chromatograms differed among the samples (Figure 1B). We identified five components (Figure 1C) that are known as common plant volatiles  and tested them for EAG activity (Figure 1C). All components generated antennal responses. In summary, the microhabitat blends were stable over time, differed between samples, and could be detected by the ants. Hence, they present potential olfactory landmarks.

### Ants learn to associate the nest entrance with environmental odours in their surroundings

**Figure 2:**
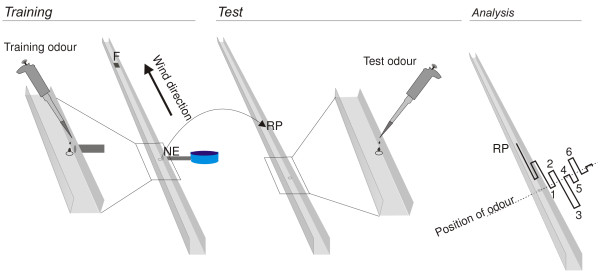
**Experimental paradigm**. *Training*. Nest situated within blue border strip; channel width and height, 7 cm, length, 16 m; position of feeder (F), 8 m downwind from nest entrance (NE); Training odour, 20 μl of either indole, nonanal, decanal, methyl salicylate (each diluted 1:50 in hexane), or hexane as solvent control. Odours were reapplied every 20 min. *Test*. Capture site of ants at NE; point of release at RP; position of odour in test channel 1 m upwind of RP; ants were tested with training odour, non-training odour, or solvent control. *Analysis*. Schematic search run. Six turning points after the ant had passed the odour for the first time were analysed for their median distance to the stimulus.

Having shown that the habitat offers potential odour-landmark information, we checked whether the ants were able to use such information for navigation. Ants were trained to forage within an open linear channel to a feeder 8 m downwind from the nest entrance (Figure 2). At the inconspicuous exit hole leading from the channel to the nest, we applied one of four mono-molecular odours (nonanal, decanal, methyl salicylate or indole) to the channel floor. Two of these odours were also present in the samples from the habitat (Figure 1), and all four showed electroantennographic activity but did not trigger any innate attraction in naïve ants (Additional file 1). Would the ants learn to associate the nest entrance with the given odour? Would they distinguish the training odour from other non-training odours? And would they be able to recognise the training odour against a background of an odour mixture? We captured homing ants at the nest entrance and released them in a test channel that was identical to the training channel in its dimensions and orientation but lacked a nest entrance. The release point was 1 m downwind of an odour stimulus that either

i) was identical to the training odour,

ii) consisted of another odour,

iii) consisted of a mixture of four odours including the training odour,

iv) consisted of a drop of the solvent hexane (solvent control).

**Figure 3:**
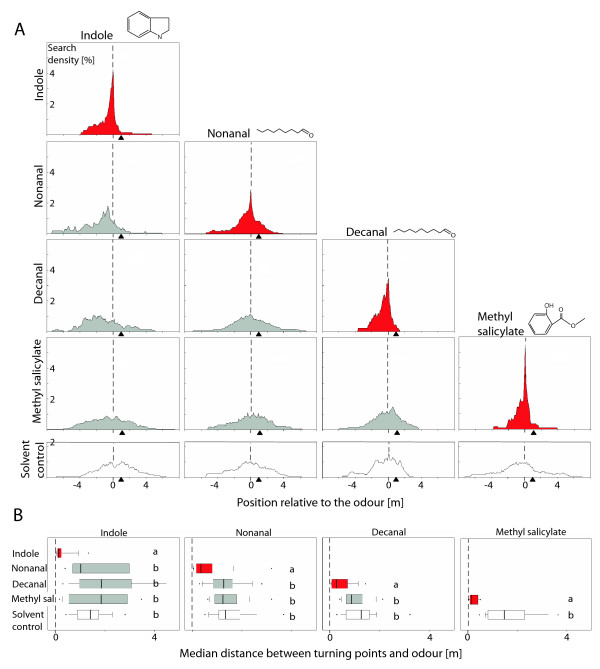
**Discrimination among odours**. A. Relative search densities of ants tested with the training odour (red plots), non-training odours (grey plots), or with the solvent as a control (white plots). Diagram columns, training odours; diagram rows, test odours; dashed line, position of odour; black arrow heads, point of release; sample size, 20 ants per plot. Search plots include the first six turning points after the ants had passed the odour for the first time. For details of graph construction see [11]. B. Median distances between the turning points and the position of odour (dashed line). Box plot: black line, median; box, interquartile range; whiskers, 10^th ^and 90^th ^percentiles, black dots, outliers. Within each plot, diagram letters indicate significant differences (p < 0.01, Dunn's post hoc test) between the groups.

In order to check whether the ants focused their search on the position of the applied odour, we recorded the turning points of the nest-searching ants in the test channel. We analysed six turning points after the ant had passed the odour for the first time for the median distance to the stimulus. The values of the differentially treated groups were tested for significant differences by Kruskal-Wallis analysis and a Dunn's post hoc test. When tested with the trained odour, the ants' search was well directed (Figure 3). However, when they were tested with non-trained odours the ants' search did not differ from that displayed when tested with the solvent control (Figure 3). Hence, the ants were able to associate each of the four odours with the nest entrance and were able to distinguish the learned odour from the non-training odours.

**Figure 4:**
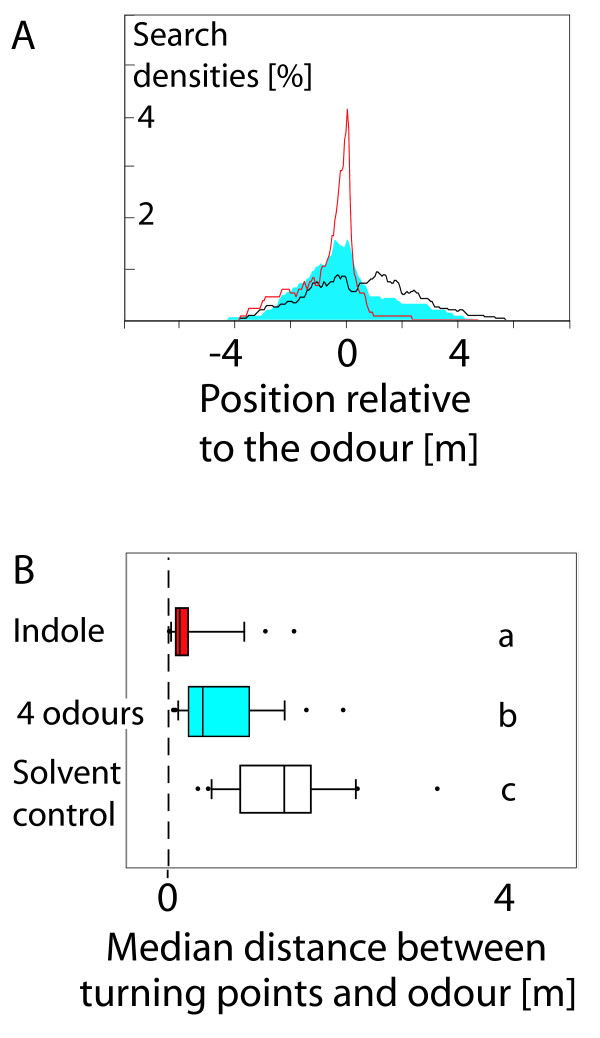
**Recognition of a learned odour in a blend**. A. Relative search densities of ants that were trained with indole and tested either with indole (red line), with the solvent (black line), or with a blend of indole, nonanal, decanal, and methyl salicylate (blue area). For details see Figure 3. B. Median distances between the turning points and the position of odour (dashed line). For details see Figure 3. Diagram letters indicate significant differences (p < 0.01, Dunn's post hoc test) between the groups.

Furthermore, when ants were trained to indole and tested with a mixture of all four odours, their search accuracy again decreased. However, in this case they were still better-directed than they were when tested with the solvent control (Figure 4). Thus, the ants were less sure about the position of the nest when the trained odour was provided in a blend during the test. However, they were still able to recognise the learned odour against the background of three additional odours.

## Discussion

Desert ants, *Cataglyphis*, are known to rely on path integration [1,2,5] and visual landmarks [3,4] during homing. In the present study, we ask whether ants associate their nest entrance with environmental odours. By collecting and analysing volatiles from different positions within the salt-pan habitat of *C. fortis*, we show that the environment provides the ants with potential odour-landmark information, i.e. with different place-specific blends (Figure 1). The blend components that were identified induced EAG responses in these ants. Can the ants make use of such information? Yes, they can. Homing ants that were trained to the nest marked by an odour focused their nest search on this odour in a test situation, but they did not search at the solvent control (Figure 3, [11]). Hence, *Cataglyphis *ants can learn the association between the nest entrance and an environmental odour and use this information for homing.

The salt-pan habitat is not an odour-free space, interrupted by single odour peaks but, rather, is loaded with a variety of odours (Figure 1). Hence, when using olfactory information as landmarks, ants must be able to distinguish a learned odour from other odours. As the ability to discriminate among odours relates to the dissimilarity among molecules, bees frequently confuse odours that share functional groups or have similar chain lengths [12]. The most similar among the four odours tested in the present account were the aldehydes nonanal and decanal; they have the same functional group and chain lengths that differ by only one carbon atom, whereas methyl salicylate and indole have different chemical structures (Figure 3A). Even so, the ants were able to discriminate among all of them (Figure 3). Ants that were tested with a non-trained odour did not avoid this odour during the nest search as naïve ants seemed to do (Additional file 1). The training on one odour resulted in an odour-specific response by homing ants, which is one prerequisite for odour-landmark navigation.

The use of odour landmarks requires a further skill: the olfactory background of a stimulus might change dramatically when for example the wind direction changes and an odour source suddenly appears upwind of the landmark. In order to use an olfactory landmark, an ant must be able to identify the learned odour against a changing background of odours. *Cataglyphis *ants fulfilled this demand when trained on indole and tested with a blend of four odours including indole. Ants that were tested with the blend showed a less focused search than ants that were tested with the trained odour only, but still a more focused search than ant tested with the solvent control (Figure 4).

Hence, the ants can use olfactory information for homing. Trail following is the predominant means of orientation in a large number of ant species [13]. These trails always consist of ant-derived trail pheromones. Unlike orientation that is guided by pheromones, orientation that uses non-pheromonal chemical cues is less well investigated. Carpenter ants can be trained to search for food on specific species of trees. Trained ants decide for the right tree species even when tactile cues are experimentally excluded. Therefore, the ants seem to use tree-derived chemical cues [14]. Bees have been shown to associate different odours with different feeding places. Blowing a learned odour into the hive triggered the trained bees to visit the corresponding feeder, i.e. navigational memory can be evoked just by providing the learned odour [15]. Finally, it has been shown that around cities, spatial gradients of atmospheric volatiles exist [16] and seem to be used by pigeons to pinpoint their loft [17]. Unlike bees and pigeons, the desert ant *Cataglyphis *has so far been a model only for visually guided orientation.

We are amazed to discover that while keeping track of the path integrator and learning visual landmarks, these ants can also collect information about the olfactory world. In future experiments, we hope to clarify how visual and olfactory landmarks interact to provide accurate information regarding, for example, nest location.

## Conclusion

The desert ant *Cataglyphis fortis *has been deemed a typical vision guided insect. Here we show that the ants' habitat exhibits location-specific blends that are stable over time and can be detected by the ants. Therefore, the environment provides potential olfactory landmarks. We also show that the ants can associate a specific place (the nest entrance) with an odour, can distinguish between learned and non-learned odours, and, finally, are able to recognize a learned odour in front of a complex blend. Hence, they can make use of the olfactory information offered by the environment to pinpoint their nest entrance.

## Methods

### Location-specific scents in the salt-pan habitat

We collected ground structures from the ants' habitat in a salt-pan close to Menzel Chaker, Tunisia, in order to analyse their odour composition. Samples of dead wood, plants, clefts and closed salt crust were brought to the laboratory within odourless oven bags. The air within each of the oven bags was exchanged for 3 litres of purified air. An empty bag served as control. The samples were put in an oven and kept at 45°C for 3 hours. The air within the bags was pumped over a thermal desorption filter containing Carbotrap B and Tenax [18]. The next day we again collected odours from the same samples under identical conditions. Headspace samples were analysed on an Agilent Technologies 7890A GC-MS system fitted with a GERSTEL Thermal Desorption Unit and equipped with an HP5ms column (30 m × 250 μm × 0.25 μm). Helium (1 ml/min constant flow) was used as a carrier gas. Samples were heated to 40°C for 5 min. Temperature was then increased at a rate of 5°/min to 200°C. After another 20 min, the temperature was further increased at a rate of 30°/min to 280° for 10 min. Dominant peaks were identified using the NIST 2005 library and were confirmed by the injection of synthetic reference compounds.

### EAG activity of identified components

We recorded EAG responses for 7 different components (5 identified habitat odours: hexanal, octanal, nonanal, camphor, decanal (Figure 1C); 2 non-habitat odours: indole, methyl salicylate [data not shown]). The basal end of the isolated antenna was inserted into a glass microelectrode filled with standard insect saline solution. The tip of the antenna was cut off and inserted into the reference electrode filled with the same saline solution. The antenna was placed in a humidified air stream (0.3 l/min, blown through a glass tube with 8 mm diameter with its end positioned about 1 cm from the antenna). We inserted a Pasteur pipette cartridge into a small hole in the tube, 10 cm from the outlet. The cartridge contained 2 μl of diluted odour (1:100 in hexane) dropped on a piece of filter paper. To deliver the odour stimulus, we puffed purified air (0.2 s at 0.1 l/min) through the odour cartridge, using a stimulus flow controller (SFC-2, Syntech^®^, The Netherlands). EAG signals from the antenna were amplified with a head-stage preamplifier (EAGPro, Syntech^®^, The Netherlands) and further processed with a PC-based signal processing system (EAGPro, Syntech^®^, The Netherlands).

### The ants' ability to use olfactory landmarks

Ants were trained in an aluminium channel open at its top to a feeder 8 m downwind of the nest entrance (Figure 2). Each ant arriving at the feeder was individually marked by a two-colour code. At the nest entrance, we dropped 20 μl of a diluted mono-molecular odour (1:50 in hexane). The odour was reapplied every 20 min to ensure an olfactory cue at any time. Training experiments were performed with nonanal, decanal, methyl salicylate and indole. In addition to nonanal and decanal that were present in the ants' habitat, we used methyl salycilate and indole because they are also common plant volatiles with high boiling points, i.e. are easy to handle under hot desert conditions. These odours elicited electroantennographic activity in *Cataglyphis fortis *(nonanal and decanal, Figure 1C, data for methyl salicylate and indole not shown) but did not generate any innate attraction in naïve ants (Additional file 1). Returning ants (with an experience of at least 15 forage runs) were captured at the nest entrance and transferred, together with a food item to ensure homing motivation, to a remote parallel test channel. The release point was situated 1 m downwind of the odour stimulus. The ants were tested either with the training odour, with one of the non-training odours, or with a blend of four odours containing the training odour. In the solvent control, the ants that were trained to one of the four odours were tested with a pure solvent stimulus. We tested 20 ants in each test situation and every animal was tested only once. As a measure of the ants' search accuracy, we used the median distance of the first six turning points from the position of the odour after the ants had passed the odour for the first time (Figure 2C).

## Competing interests

The authors declare that they have no competing interests.

## Authors' contributions

KS and MK designed and carried out the experiments and performed statistical analysis. All authors were involved in interpreting the results and writing the manuscript. All authors read and approved the final manuscript.

## Supplementary Material

Additional file 1 **Response of naïve ants to the study odours.** The data provided give evidence that the odours used in this study are not innately attractive to *Cataglyphis*. Click here for file
