# Supplementary material for: Smells like home: Desert ants, Cataglyphis fortis, use olfactory landmarks to pinpoint the nest
Source: Front Zool. 2009 Feb 27;6:5. doi: 10.1186/1742-9994-6-5 (PMC2651142; doi:10.1186/1742-9994-6-5)
Supplement: Additional file 1 — Response of naïve ants to the study odours. The data provided give evidence that the odours used in this study are not innately attractive to Cataglyphis. [file 1742-9994-6-5-S1.doc]

###
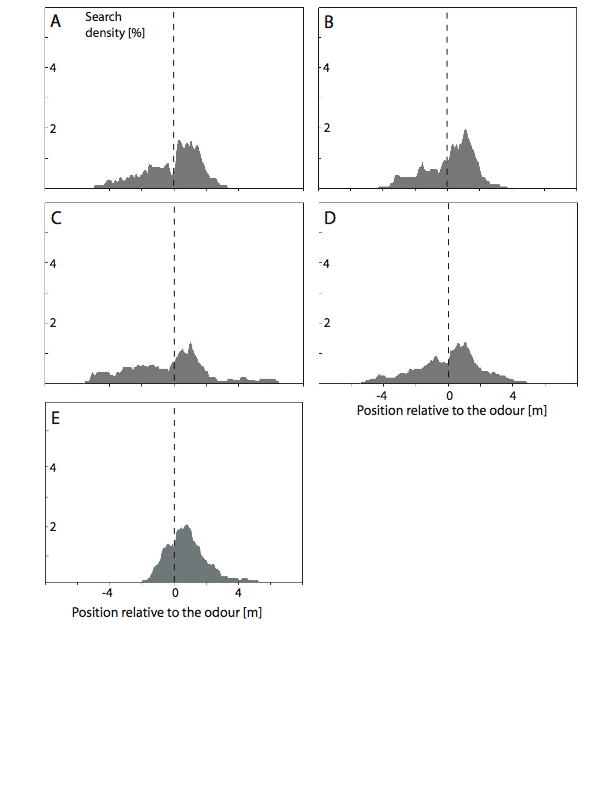
Additional Fig 1 – Response of naïve ants to the study odours.

A. Indole, B. Nonanal, C. Decanal, D. Methyl salicylate, E. Blend of the four components. Dashed line, position of odour; black arrowheads, point of release; sample size, 20 ants per plot. Search plots include the first 6 turning points after the ants had passed the odour for the first time.
